# (2,2′-Bipyridine-κ^2^ *N*,*N*′){*N*-[2-oxido-5-(phenyl­diazen­yl)benzyl­idene-κ*O*]glycinato-κ^2^ *N*,*O*}copper(II)

**DOI:** 10.1107/S1600536809036423

**Published:** 2009-09-16

**Authors:** Qiu-Xia Zhang, Gan-Qing Zhao, Jian-Qi Zhu, Ling-Wei Xue, Yong-Jun Han

**Affiliations:** a School of Chemistry and Chemical Engineering, Pingdingshan University, Pingdingshan 467000, People’s Republic of China

## Abstract

In the title compound, [Cu(C_15_H_11_N_3_O_3_)(C_10_H_8_N_2_)], the Cu^II^ atom is five-coordinated in a distorted square-pyramidal CuN_3_O_2_ geometry. The basal positions are occupied by three donor atoms from the tridentate Schiff base ligand and by one N atom from the 2,2′-bipyridine ligand. The axial position is occupied by the other N atom of the 2,2′-bipyridine ligand. The crystal structure is consolidated by weak C—H⋯O hydrogen bonds. In addition, π–π inter­actions between adjacent pyridine rings (centroid–centroid distances = 3.238 and 3.313 Å) may also stabilize the crystal packing.

## Related literature

For related structures of copper(II) with Schiff base ligands, see: Raso *et al.* (1996 ▶, 1999 ▶); Reddy *et al.* (2002 ▶); Wang *et al.* (2005 ▶); Warda (1997 ▶, 1998*a* ▶,*b* ▶,*c* ▶). For the synthesis of the ligand, see: Wei *et al.* (2007 ▶). For the synthesis of the title compound, see: Plesch *et al.* (1997 ▶). 
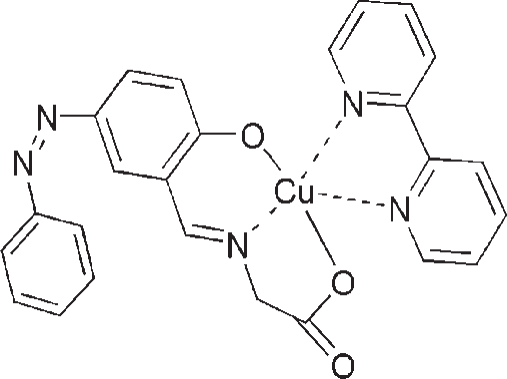


## Experimental

### 

#### Crystal data

[Cu(C_15_H_11_N_3_O_3_)(C_10_H_8_N_2_)]
*M* *_r_* = 500.99
Monoclinic, 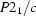

*a* = 12.604 (3) Å
*b* = 12.487 (3) Å
*c* = 13.962 (3) Å
β = 93.05 (3)°
*V* = 2194.3 (9) Å^3^
*Z* = 4
Mo *K*α radiation
μ = 1.03 mm^−1^
*T* = 296 K
0.20 × 0.20 × 0.20 mm

#### Data collection

Bruker SMART APEXII CCD diffractometer
Absorption correction: multi-scan (*SADABS*; Sheldrick, 1996 ▶) *T*_min_ = 0.820, *T*_max_ = 0.820
11072 measured reflections
3882 independent reflections
3331 reflections with *I* > 2σ(*I*)
*R*_int_ = 0.020

#### Refinement

*R*[*F* ^2^ > 2σ(*F* ^2^)] = 0.027
*wR*(*F* ^2^) = 0.074
*S* = 1.07
3882 reflections
307 parameters
H-atom parameters constrained
Δρ_max_ = 0.21 e Å^−3^
Δρ_min_ = −0.36 e Å^−3^

### 

Data collection: *APEX2* (Bruker, 2008 ▶); cell refinement: *SAINT* (Bruker, 2008 ▶); data reduction: *SAINT*; program(s) used to solve structure: *SHELXS97* (Sheldrick, 2008 ▶); program(s) used to refine structure: *SHELXL97* (Sheldrick, 2008 ▶); molecular graphics: *SHELXTL* (Sheldrick, 2008 ▶); software used to prepare material for publication: *SHELXTL*.

## Supplementary Material

Crystal structure: contains datablocks global, I. DOI: 10.1107/S1600536809036423/wm2250sup1.cif

Structure factors: contains datablocks I. DOI: 10.1107/S1600536809036423/wm2250Isup2.hkl

Additional supplementary materials:  crystallographic information; 3D view; checkCIF report

## Figures and Tables

**Table 1:** Selected bond lengths (Å)

Cu1—O1	1.9303 (15)
Cu1—N3	1.9354 (17)
Cu1—O2	1.9501 (15)
Cu1—N1	2.0282 (17)
Cu1—N2	2.2392 (18)

**Table 2:** Hydrogen-bond geometry (Å, °)

*D*—H⋯*A*	*D*—H	H⋯*A*	*D*⋯*A*	*D*—H⋯*A*
C25—H25⋯O3^i^	0.93	2.56	3.250 (3)	131
C12—H12*B*⋯O3^ii^	0.97	2.44	3.365 (3)	160
C4—H4⋯O2^iii^	0.93	2.50	3.082 (3)	121

Symmetry codes: (i) 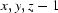; (ii) 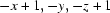; (iii) 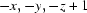.

**Fig. 1.**
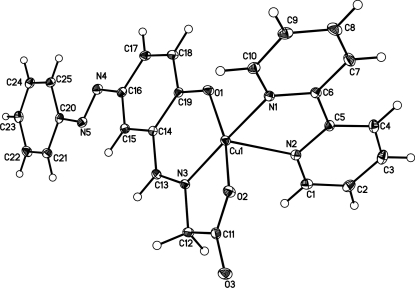
The structure of the title compound, showing 30% probability displacement ellipsoids and the atom-numbering scheme.

**Fig. 2.**
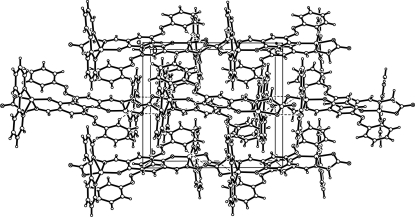
A view of the crystal packing along the a axis. Hydrogen bonds are shown as dashed lines.

## supplementary crystallographic information

### Comment

Considerable efforts have been devoted to copper(II) complexes of tridentate Schiff base ligands of the *N*-alkylidene or *N*-arylidene aminoacidato type. These compound are interesting in terms of their structural varieties, their electrochemical properties as well as their nature as potential model substances for a number of important biological systems (Raso *et al.*, 1996, 1999). Several stuctural studies have been performed on Schiff base copper(II) complexes derived from salicylaldehyde and animo acids (Reddy *et al.*, 2002; Wang *et al.*, 2005; Warda, 1997, 1998*a,b,c*). We report here the crystal structure of the title Cu^II^ complex.

The structure consists of a discrete monomeric square-pyramidal Cu^II^ complex (Fig. 1 and Table 1). The basal positions are occupied by three donor atoms from the tridentate Schiff base ligand, which furnishes an ONO donor set, with the fourth position occupied by one N atom from the 2, 2'-bipyridine ligand. The axial position is occupied by the other N atom of the 2, 2'-bipyridine igand. The Cu atom is displaced from the O1/O2/N1/N3 basal plane toward the N2 atom by -0.0169 Å.

The 2,2'-bipyridine ligand and the benzene ring (C20···C25) are essentially planar and form a dihedral angle of 92.5° and 15.8°, respectively, with the benzene ring of the Schiff base ligand.

The crystal structure is stabilized by weak hydrogen bonds, such as C4—H4···O2 (3.082 Å), C3—H3···O1 (3.365 Å) and C25—H25···O3 (3.250 Å). The π-π interactions between the adjacent pyridine rings [C1···C9 (3.238 Å) and C2···C10 (3.313 Å)] play important role in the stability of the complex (Fig. 2 and Table 2).

### Experimental

The compound of 5-(phenyldiazenyl)salicylaldehyde was synthesized as described in the literature (Wei *et al.*, 2007). The title compound was synthesized as described in the literature (Plesch *et al.*, 1997): To glycine (1.00 mmol) and potassium hydroxide (1.00 mmol) in 10 ml of methanol was added 5-(phenyldiazenyl)salicylaldehyde (1.00 mmol) in 30 ml of dimethylfomamide dropwise. The yellowish-brown solution was stirred for 2.0 h at 333 K. The resultant mixture was added dropwise to copper(II) acetate monohydrate (1.00 mmol) and 2, 2'-bipyridine (1.00 mmol) in 10 ml of methanol, and heated with stirring for 2.0 h at 333 K. The dark green solution was filtered and left for several days. Dark-green crystals had formed that were filtered off, washed with water, and dried under vacuum.

### Refinement

In the title compound, all H atoms were positioned geometrically and refined as riding atoms, with C—H = 0.93 (CH) and 0.97 Å (CH_2_) and *U*_iso_(H) = 1.2*U*_eq_(C).

### Crystal data

**Table:**

[Cu(C_15_H_11_N_3_O_3_)(C_10_H_8_N_2_)]	*F*(000) = 1028
*M**_r_* = 500.99	*D*_x_ = 1.516 Mg m^−^^3^
Monoclinic, *P*2_1_/*c*	Mo *K*α radiation, λ = 0.71073 Å
Hall symbol: -P 2ybc	Cell parameters from 5614 reflections
*a* = 12.604 (3) Å	θ = 2.2–27.5°
*b* = 12.487 (3) Å	µ = 1.03 mm^−^^1^
*c* = 13.962 (3) Å	*T* = 296 K
β = 93.05 (3)°	Block, dark green
*V* = 2194.3 (9) Å^3^	0.20 × 0.20 × 0.20 mm
*Z* = 4	

### Data collection

**Table:**

Bruker SMART APEXII CCD diffractometer	3882 independent reflections
Radiation source: fine-focus sealed tube	3331 reflections with *I* > 2σ(*I*)
graphite	*R*_int_ = 0.020
ω scans	θ_max_ = 25.1°, θ_min_ = 2.2°
Absorption correction: multi-scan (*SADABS*; Sheldrick, 1996)	*h* = −15→10
*T*_min_ = 0.820, *T*_max_ = 0.820	*k* = −14→14
11072 measured reflections	*l* = −16→16

### Refinement

**Table:**

Refinement on *F*^2^	Primary atom site location: structure-invariant direct methods
Least-squares matrix: full	Secondary atom site location: difference Fourier map
*R*[*F*^2^ > 2σ(*F*^2^)] = 0.027	Hydrogen site location: inferred from neighbouring sites
*wR*(*F*^2^) = 0.074	H-atom parameters constrained
*S* = 1.07	*w* = 1/[σ^2^(*F*_o_^2^) + (0.0343*P*)^2^ + 0.837*P*] where *P* = (*F*_o_^2^ + 2*F*_c_^2^)/3
3882 reflections	(Δ/σ)_max_ = 0.001
307 parameters	Δρ_max_ = 0.21 e Å^−^^3^
0 restraints	Δρ_min_ = −0.36 e Å^−^^3^

### Special details

**Table:**

Geometry. All e.s.d.'s (except the e.s.d. in the dihedral angle between two l.s. planes) are estimated using the full covariance matrix. The cell e.s.d.'s are taken into account individually in the estimation of e.s.d.'s in distances, angles and torsion angles; correlations between e.s.d.'s in cell parameters are only used when they are defined by crystal symmetry. An approximate (isotropic) treatment of cell e.s.d.'s is used for estimating e.s.d.'s involving l.s. planes.
Refinement. Refinement of *F*^2^ against ALL reflections. The weighted *R*-factor *wR* and goodness of fit *S* are based on *F*^2^, conventional *R*-factors *R* are based on *F*, with *F* set to zero for negative *F*^2^. The threshold expression of *F*^2^ > σ(*F*^2^) is used only for calculating *R*-factors(gt) *etc*. and is not relevant to the choice of reflections for refinement. *R*-factors based on *F*^2^ are statistically about twice as large as those based on *F*, and *R*- factors based on ALL data will be even larger.

### Fractional atomic coordinates and isotropic or equivalent isotropic displacement parameters (Å^2^)

**Table:**

	*x*	*y*	*z*	*U*_iso_*/*U*_eq_	
Cu1	0.192784 (18)	−0.04691 (2)	0.315857 (17)	0.03322 (9)	
C1	0.09307 (19)	0.18579 (18)	0.36777 (16)	0.0465 (5)	
H1	0.1606	0.2138	0.3609	0.056*	
C2	0.0121 (2)	0.2551 (2)	0.38779 (17)	0.0522 (6)	
H2	0.0247	0.3282	0.3942	0.063*	
C3	−0.0875 (2)	0.2136 (2)	0.39801 (17)	0.0555 (6)	
H3	−0.1437	0.2586	0.4112	0.067*	
C4	−0.10356 (18)	0.1052 (2)	0.38851 (16)	0.0469 (6)	
H4	−0.1706	0.0759	0.3953	0.056*	
C5	−0.01873 (16)	0.04031 (17)	0.36876 (14)	0.0356 (5)	
C6	−0.02777 (16)	−0.07837 (17)	0.36193 (14)	0.0367 (5)	
C7	−0.12214 (18)	−0.1328 (2)	0.37705 (17)	0.0513 (6)	
H7	−0.1840	−0.0951	0.3881	0.062*	
C8	−0.1224 (2)	−0.2431 (2)	0.37532 (18)	0.0571 (7)	
H8	−0.1848	−0.2804	0.3847	0.069*	
C9	−0.0307 (2)	−0.29785 (19)	0.35978 (16)	0.0493 (6)	
H9	−0.0295	−0.3723	0.3599	0.059*	
C10	0.05944 (18)	−0.23985 (17)	0.34394 (14)	0.0414 (5)	
H10	0.1218	−0.2767	0.3329	0.050*	
C11	0.32869 (16)	−0.00987 (18)	0.47223 (15)	0.0380 (5)	
C12	0.38063 (17)	0.05212 (17)	0.39264 (14)	0.0386 (5)	
H12A	0.3773	0.1284	0.4055	0.046*	
H12B	0.4548	0.0318	0.3907	0.046*	
C13	0.36753 (16)	0.04938 (16)	0.22099 (14)	0.0344 (5)	
H13	0.4359	0.0775	0.2242	0.041*	
C14	0.31658 (16)	0.03250 (15)	0.12687 (14)	0.0321 (4)	
C15	0.37072 (17)	0.06897 (17)	0.04804 (14)	0.0368 (5)	
H15	0.4385	0.0977	0.0582	0.044*	
C16	0.32614 (17)	0.06347 (16)	−0.04450 (14)	0.0380 (5)	
C17	0.22613 (18)	0.01671 (18)	−0.05992 (15)	0.0436 (5)	
H17	0.1961	0.0110	−0.1220	0.052*	
C18	0.17160 (18)	−0.02104 (18)	0.01584 (15)	0.0414 (5)	
H18	0.1052	−0.0521	0.0037	0.050*	
C19	0.21339 (16)	−0.01413 (16)	0.11159 (14)	0.0329 (4)	
C20	0.50636 (17)	0.19390 (17)	−0.19420 (14)	0.0380 (5)	
C21	0.59128 (18)	0.26270 (19)	−0.17756 (16)	0.0474 (6)	
H21	0.6141	0.2804	−0.1151	0.057*	
C22	0.64230 (19)	0.3052 (2)	−0.25454 (18)	0.0547 (6)	
H22	0.6985	0.3527	−0.2438	0.066*	
C23	0.60982 (19)	0.2772 (2)	−0.34675 (17)	0.0512 (6)	
H23	0.6447	0.3053	−0.3982	0.061*	
C24	0.52609 (19)	0.20795 (19)	−0.36326 (16)	0.0469 (6)	
H24	0.5049	0.1891	−0.4258	0.056*	
C25	0.47297 (18)	0.16593 (18)	−0.28733 (15)	0.0426 (5)	
H25	0.4158	0.1197	−0.2986	0.051*	
N1	0.06132 (13)	−0.13269 (13)	0.34377 (11)	0.0337 (4)	
N2	0.07904 (14)	0.08041 (14)	0.35774 (12)	0.0387 (4)	
N3	0.32500 (13)	0.02827 (13)	0.30014 (11)	0.0322 (4)	
N4	0.37471 (15)	0.10262 (15)	−0.12741 (12)	0.0440 (4)	
N5	0.45784 (15)	0.15498 (15)	−0.11024 (12)	0.0421 (4)	
O1	0.15568 (11)	−0.04915 (11)	0.17994 (10)	0.0393 (3)	
O2	0.25223 (12)	−0.07232 (13)	0.44568 (10)	0.0437 (4)	
O3	0.36149 (13)	0.00378 (16)	0.55548 (11)	0.0560 (4)	

### Atomic displacement parameters (Å^2^)

**Table:**

	*U*^11^	*U*^22^	*U*^33^	*U*^12^	*U*^13^	*U*^23^
Cu1	0.02967 (15)	0.03941 (16)	0.03078 (14)	−0.00550 (10)	0.00342 (10)	0.00006 (10)
C1	0.0491 (14)	0.0435 (14)	0.0464 (13)	−0.0057 (11)	−0.0006 (10)	−0.0030 (10)
C2	0.0697 (17)	0.0393 (13)	0.0475 (13)	0.0076 (12)	0.0005 (12)	−0.0042 (10)
C3	0.0613 (17)	0.0569 (16)	0.0491 (14)	0.0210 (13)	0.0113 (12)	0.0023 (12)
C4	0.0420 (13)	0.0571 (16)	0.0426 (13)	0.0055 (11)	0.0105 (10)	0.0074 (11)
C5	0.0351 (11)	0.0443 (12)	0.0275 (10)	0.0011 (9)	0.0040 (8)	0.0025 (9)
C6	0.0364 (11)	0.0469 (12)	0.0267 (10)	−0.0055 (9)	0.0024 (8)	0.0019 (9)
C7	0.0367 (12)	0.0621 (16)	0.0558 (14)	−0.0076 (11)	0.0098 (11)	0.0021 (12)
C8	0.0506 (15)	0.0612 (17)	0.0600 (16)	−0.0252 (13)	0.0065 (12)	0.0030 (13)
C9	0.0635 (16)	0.0422 (13)	0.0423 (13)	−0.0164 (12)	0.0023 (11)	0.0004 (10)
C10	0.0484 (13)	0.0409 (13)	0.0350 (11)	−0.0052 (10)	0.0031 (10)	−0.0009 (9)
C11	0.0321 (11)	0.0486 (13)	0.0333 (11)	0.0042 (10)	0.0031 (9)	0.0010 (9)
C12	0.0359 (11)	0.0458 (13)	0.0338 (11)	−0.0063 (9)	−0.0017 (9)	−0.0021 (9)
C13	0.0293 (10)	0.0367 (11)	0.0374 (11)	−0.0036 (9)	0.0033 (9)	0.0020 (9)
C14	0.0327 (11)	0.0314 (11)	0.0324 (10)	0.0018 (8)	0.0036 (8)	0.0009 (8)
C15	0.0353 (11)	0.0395 (12)	0.0358 (11)	−0.0046 (9)	0.0055 (9)	−0.0006 (9)
C16	0.0456 (13)	0.0366 (12)	0.0323 (11)	−0.0002 (9)	0.0072 (9)	0.0001 (9)
C17	0.0490 (14)	0.0506 (14)	0.0307 (11)	−0.0055 (11)	−0.0007 (10)	−0.0018 (9)
C18	0.0416 (12)	0.0456 (13)	0.0366 (11)	−0.0094 (10)	−0.0010 (9)	−0.0017 (9)
C19	0.0351 (11)	0.0295 (10)	0.0343 (11)	0.0004 (8)	0.0037 (9)	−0.0021 (8)
C20	0.0416 (12)	0.0392 (12)	0.0340 (11)	0.0052 (9)	0.0085 (9)	0.0012 (9)
C21	0.0454 (13)	0.0555 (15)	0.0413 (12)	−0.0025 (11)	0.0034 (10)	−0.0030 (11)
C22	0.0415 (13)	0.0637 (16)	0.0594 (16)	−0.0094 (12)	0.0083 (11)	0.0035 (12)
C23	0.0496 (14)	0.0568 (15)	0.0488 (14)	0.0030 (12)	0.0178 (11)	0.0109 (11)
C24	0.0580 (15)	0.0499 (14)	0.0334 (11)	0.0053 (11)	0.0083 (10)	0.0026 (10)
C25	0.0471 (13)	0.0427 (12)	0.0385 (12)	−0.0026 (10)	0.0054 (10)	−0.0002 (10)
N1	0.0341 (9)	0.0380 (10)	0.0291 (8)	−0.0038 (7)	0.0028 (7)	0.0005 (7)
N2	0.0374 (10)	0.0401 (10)	0.0387 (10)	−0.0021 (8)	0.0028 (8)	−0.0028 (8)
N3	0.0300 (9)	0.0365 (10)	0.0299 (9)	−0.0018 (7)	0.0002 (7)	0.0007 (7)
N4	0.0503 (11)	0.0494 (11)	0.0329 (9)	−0.0043 (9)	0.0081 (8)	0.0009 (8)
N5	0.0479 (11)	0.0448 (11)	0.0343 (10)	−0.0015 (9)	0.0087 (8)	0.0003 (8)
O1	0.0357 (8)	0.0498 (9)	0.0325 (7)	−0.0130 (7)	0.0039 (6)	0.0001 (6)
O2	0.0373 (8)	0.0599 (10)	0.0339 (8)	−0.0103 (7)	0.0030 (6)	0.0062 (7)
O3	0.0504 (10)	0.0857 (13)	0.0313 (8)	−0.0114 (9)	−0.0034 (7)	0.0015 (8)

### Geometric parameters (Å, °)

**Table:**

Cu1—O1	1.9303 (15)	C12—H12A	0.9700
Cu1—N3	1.9354 (17)	C12—H12B	0.9700
Cu1—O2	1.9501 (15)	C13—N3	1.281 (3)
Cu1—N1	2.0282 (17)	C13—C14	1.447 (3)
Cu1—N2	2.2392 (18)	C13—H13	0.9300
C1—N2	1.334 (3)	C14—C15	1.402 (3)
C1—C2	1.377 (3)	C14—C19	1.431 (3)
C1—H1	0.9300	C15—C16	1.383 (3)
C2—C3	1.373 (3)	C15—H15	0.9300
C2—H2	0.9300	C16—C17	1.395 (3)
C3—C4	1.375 (4)	C16—N4	1.424 (3)
C3—H3	0.9300	C17—C18	1.375 (3)
C4—C5	1.381 (3)	C17—H17	0.9300
C4—H4	0.9300	C18—C19	1.413 (3)
C5—N2	1.347 (3)	C18—H18	0.9300
C5—C6	1.489 (3)	C19—O1	1.306 (2)
C6—N1	1.347 (3)	C20—C21	1.382 (3)
C6—C7	1.395 (3)	C20—C25	1.390 (3)
C7—C8	1.378 (4)	C20—N5	1.435 (3)
C7—H7	0.9300	C21—C22	1.387 (3)
C8—C9	1.370 (4)	C21—H21	0.9300
C8—H8	0.9300	C22—C23	1.375 (3)
C9—C10	1.375 (3)	C22—H22	0.9300
C9—H9	0.9300	C23—C24	1.375 (3)
C10—N1	1.338 (3)	C23—H23	0.9300
C10—H10	0.9300	C24—C25	1.387 (3)
C11—O3	1.225 (2)	C24—H24	0.9300
C11—O2	1.279 (3)	C25—H25	0.9300
C11—C12	1.530 (3)	N4—N5	1.247 (2)
C12—N3	1.467 (3)		
			
O1—Cu1—N3	93.47 (7)	C14—C13—H13	117.7
O1—Cu1—O2	166.50 (7)	C15—C14—C19	119.47 (18)
N3—Cu1—O2	83.90 (7)	C15—C14—C13	117.05 (18)
O1—Cu1—N1	91.35 (6)	C19—C14—C13	123.42 (17)
N3—Cu1—N1	174.37 (7)	C16—C15—C14	121.80 (19)
O2—Cu1—N1	90.74 (7)	C16—C15—H15	119.1
O1—Cu1—N2	98.17 (7)	C14—C15—H15	119.1
N3—Cu1—N2	104.62 (7)	C15—C16—C17	118.92 (19)
O2—Cu1—N2	95.31 (7)	C15—C16—N4	124.85 (19)
N1—Cu1—N2	77.52 (7)	C17—C16—N4	116.23 (19)
N2—C1—C2	123.0 (2)	C18—C17—C16	120.6 (2)
N2—C1—H1	118.5	C18—C17—H17	119.7
C2—C1—H1	118.5	C16—C17—H17	119.7
C3—C2—C1	118.4 (2)	C17—C18—C19	122.0 (2)
C3—C2—H2	120.8	C17—C18—H18	119.0
C1—C2—H2	120.8	C19—C18—H18	119.0
C2—C3—C4	119.5 (2)	O1—C19—C18	118.48 (18)
C2—C3—H3	120.3	O1—C19—C14	124.38 (18)
C4—C3—H3	120.3	C18—C19—C14	117.13 (18)
C3—C4—C5	119.1 (2)	C21—C20—C25	120.44 (19)
C3—C4—H4	120.5	C21—C20—N5	115.65 (19)
C5—C4—H4	120.5	C25—C20—N5	123.9 (2)
N2—C5—C4	121.9 (2)	C20—C21—C22	119.6 (2)
N2—C5—C6	115.48 (18)	C20—C21—H21	120.2
C4—C5—C6	122.6 (2)	C22—C21—H21	120.2
N1—C6—C7	120.6 (2)	C23—C22—C21	120.1 (2)
N1—C6—C5	116.83 (17)	C23—C22—H22	120.0
C7—C6—C5	122.5 (2)	C21—C22—H22	120.0
C8—C7—C6	119.0 (2)	C24—C23—C22	120.3 (2)
C8—C7—H7	120.5	C24—C23—H23	119.9
C6—C7—H7	120.5	C22—C23—H23	119.9
C9—C8—C7	120.1 (2)	C23—C24—C25	120.5 (2)
C9—C8—H8	120.0	C23—C24—H24	119.7
C7—C8—H8	120.0	C25—C24—H24	119.7
C8—C9—C10	118.3 (2)	C24—C25—C20	119.1 (2)
C8—C9—H9	120.9	C24—C25—H25	120.5
C10—C9—H9	120.9	C20—C25—H25	120.5
N1—C10—C9	122.8 (2)	C10—N1—C6	119.21 (18)
N1—C10—H10	118.6	C10—N1—Cu1	122.89 (15)
C9—C10—H10	118.6	C6—N1—Cu1	117.90 (14)
O3—C11—O2	124.8 (2)	C1—N2—C5	118.15 (19)
O3—C11—C12	118.9 (2)	C1—N2—Cu1	130.14 (15)
O2—C11—C12	116.29 (18)	C5—N2—Cu1	111.61 (14)
N3—C12—C11	109.50 (17)	C13—N3—C12	121.03 (17)
N3—C12—H12A	109.8	C13—N3—Cu1	126.86 (14)
C11—C12—H12A	109.8	C12—N3—Cu1	111.89 (12)
N3—C12—H12B	109.8	N5—N4—C16	114.62 (18)
C11—C12—H12B	109.8	N4—N5—C20	114.24 (18)
H12A—C12—H12B	108.2	C19—O1—Cu1	126.76 (13)
N3—C13—C14	124.59 (18)	C11—O2—Cu1	114.61 (13)
N3—C13—H13	117.7		

### Hydrogen-bond geometry (Å, °)

**Table:**

*D*—H···*A*	*D*—H	H···*A*	*D*···*A*	*D*—H···*A*
C25—H25···O3^i^	0.93	2.56	3.250 (3)	131
C12—H12B···O3^ii^	0.97	2.44	3.365 (3)	160
C4—H4···O2^iii^	0.93	2.50	3.082 (3)	121

Symmetry codes: (i) *x*, *y*, *z*−1; (ii) −*x*+1, −*y*, −*z*+1; (iii) −*x*, −*y*, −*z*+1.
